# The Combination of a Biocontrol Agent *Trichoderma* *asperellum* SC012 and Hymexazol Reduces the Effective Fungicide Dose to Control Fusarium Wilt in Cowpea

**DOI:** 10.3390/jof7090685

**Published:** 2021-08-25

**Authors:** Chongyuan Zhang, Weiwei Wang, Ming Xue, Zhen Liu, Qinman Zhang, Jumei Hou, Mengyu Xing, Rui Wang, Tong Liu

**Affiliations:** 1 Key Laboratory of Green Prevention and Control of Tropical Diseases and Pests, Ministry of Education, Hainan University, Haikou 570228, China; chongyuan_zhang@outlook.com (C.Z.); 18090401210007@hainanu.edu.cn (M.X.); liuzhenhenan@163.com (Z.L.); zhangqinman1115@163.com (Q.Z.); amyliutong@163.com (J.H.); xmy93@163.com (M.X.); wangrui01093322@163.com (R.W.); 2 Key Laboratory of Genetics and Germplasm Innovation of Tropical Special Forest Trees and Ornamental Plants, Ministry of Education, Hainan University, Haikou 570228, China; wvivi2@163.com; 3 Key Laboratory of Germplasm Resources of Tropical Special Ornamental Plants of Hainan Province, College of Forestry, Hainan University, Haikou 570228, China; 4 Engineering Center of Agricultural Microbial Preparation Research and Development of Hainan, Hainan University, Haikou 570228, China

**Keywords:** *Trichoderma asperellum*, hymexazol, Fusarium wilt, cowpea

## Abstract

The use of synthetic fungicide needs to be gradually reduced because of its adverse effect on human health and the environment. An integrated approach combining fungicides with biological control agents (BCAs) can be used to reduce the fungicide doses, thereby minimizing the risks associated with chemical fungicides. In this study, the combined application of a BCA *Trichoderma* and a fungicide hymexazol was used to manage the cowpea wilt disease caused by *Fusarium* *oxysporum*. The *Trichoderma* SC012 strain, which is resistant to hymexazol, was screened out and identified as *T. asperellum*. *T. asperellum* SC012 showed hyperparasitism to *F. oxysporum* and could penetrate and encircle the hyphae of pathogen on a medium amended or not with hymexazol. When combined with hymexazol, the population density in the rhizosphere soil of cowpea showed no significant difference compared with the treatment *Trichoderma* used alone. When the concentration of *T. asperellum* SC012 or hymexazol was halved, their combined application could control cowpea wilt disease more effectively than their individual use. The findings showed that the combination of *Trichoderma* and hymexazol could reduce the use of chemical fungicide, which is eco-friendly and may be an important part of integrated control of Fusarium wilt in cowpea.

## 1. Introduction

Cowpea (*Vigna unguiculata* (Linn.) Walp.), which is rich in proteins and some important amino acids such as tryptophan and lysine, can be used as a vegetable for human beings and forage to feed livestock [1]. It is an important leguminous crop and plays a crucial part in supporting soil conservation, and facilitating symbiotic nitrogen fixation, which is compatible with integrated farming systems [2]. According to statistics, about 14.5 million hectares of land worldwide are planted with cowpeas every year. However, many biotic and abiotic factors affect the growth of cowpea [3], among which wilt disease caused by *Fusarium oxysporum* poses a great threat to cowpea production and causes yield loss ranging from 30% to 100% worldwide every year [4].

*F. oxysporum* f. sp. *tracheiphilum*, the pathogen of cowpea wilt disease, enters the plant through the root system and invades vascular tissue. The infected plant shows leaf fading, wilting, vascular bundle discoloration, and final death [5]. The thick-walled chlamydospore produced by *F. oxysporum* can survive for several years in soil [6], which makes Fusarium wilt difficult to control. Soil sterilization with fumigants such as methyl bromide is an effective method to control Fusarium wilt. However, the use of methyl bromide causes serious pollution to the environment and may lead to the development of drug resistant pathogens, and methyl bromide has banned in different countries, including EU and China. The breeding of resistant varieties is also an effective way to control the disease, but there is always a risk that the pathogen strain will evolve to overcome resistance [7]. Using of biocontrol agents for the management of plant pathogens is an eco-friendly and safe approach. *Trichoderma*, one of the most widely used biocontrol fungi found in various habitats [8], has been used to control wilt disease caused by *F. oxysporum* on many crops [9,10,11,12]. However, little attention has been paid to the efficacy of *Trichoderma* in controlling cowpea wilt disease.

*Trichoderma* performs a variety of antagonistic mechanisms against plant pathogens, such as lytic enzymes, antifungal secondary metabolites, mycoparasitism and competition for nutrients [13,14,15,16]. Meanwhile, *Trichoderma* could induce plant resistance to disease, and promote root development and growth of plants [17]. Therefore, *Trichoderma* has a wide range of agricultural uses such as being a biofertilizer, biopesticide and bioremediation agent [14,18]. A recent research showed that *Trichoderma* could penetrate and encircle the hyphae of pathogens, thus degrading and killing the pathogens [19]. 

Several species of *Trichoderma* have been used for the management of plant pathogens. However, the control effect of biocontrol fungus alone to prevent disease is slow and unstable. It may be due to the fact that biocontrol fungi, as exotic microorganisms, are easily affected by biotic and abiotic factors and are not easy to propagate and function in the soil [20]. Thus, the combination of biocontrol agent with resistance inducer or even conventional fungicide can be used to control plant disease effectively and has attracted researchers’ attention [21,22,23]. Combined use of biocontrol agent and an effective fungicide is also useful in reducing the fungicide dose to control plant pathogens, thus helping in minimizing the risks associated with chemical pesticides.

Hymexazol (3-hydroxy-5-methylisoxazole, C_4_H_5_NO_2_), which is a systemic fungicide and soil disinfectant, can inhibit the spore germination of pathogens by combining aluminum and iron ions in the soil under acidic conditions [22], and has been used to control Fusarium wilt in many crops, such as watermelon, cucumber, soybean, and so on. Hymexazol can be absorbed directly by plant roots, transferred quickly to multiple parts of the plant, and then transformed into two glucosides (O-glucoside and N-glucoside). The O-glucoside has fungitoxicity activity as a result of interference with RNA and DNA synthesis, while the N-glucoside is associated with plant growth promoting effects [24,25].

In this study, the effectiveness of the combined application of *T. asperellum* SC012 and hymexazol on cowpea Fusarium wilt was explored, with the main goal of reducing fungicide dose.

## 2. Materials and Methods

### 2.1. Fungal Isolates and Pesticide

The *Trichoderma* strains (SC012, LS007-21, HN082102.1, HL167 and DQ1) and *F. oxysporum* f. sp. *tracheiphilum* strain FC018 used in this experiment were provided by the Key Laboratory of Green Prevention and Control of Tropical Diseases and Pests (College of plant protection, Hainan University, Haikou, China). The pesticide used in the study was the 98% hymexazol technical (3-Hydroxy-5-methylisoxazole) (Weifang Huanuo Biotechnology Co., Ltd., Weifang, China).

### 2.2. Sensitivity Test of Trichoderma Strains and F. oxysporum to Hymexazol

To test the sensitivity of fungi to hymexazol, EC_50_ (concentration for 50% of maximal effect) were calculated using the method of mycelium growth rate. Strains of *Trichoderma* and *F. oxysporum* were cultured on PDA medium mixed with different concentrations of hymexazol (50, 100, 200, 300, 400 and 600 µg mL^−1^) at 28 °C, and hymexazol -free PDA medium was used as control [26]. When the fungal colony in control completely covered the Petri dishes (2 days for *Trichoderma* and 7 days for *F. oxysporum*), the diameters of colonies were measured, and the inhibition rate was calculated as follows: inhibition rate (%) = ((colony diameter of control—colony diameter of experimental group)/colony diameter of control) × 100%.

### 2.3. Taxonomic Classification of Hymexazol-Tolerant Trichoderma

The hymexazol-tolerant *Trichoderma* strain was identified on a morphological and molecular basis. For morphology identification, hyphal morphology and colony growth patterns of the *Trichoderma* strain was observed after 3, 4, 5, 6, and 7 days cultured on PDA corn meal dextrose agar (CMD: 40 g L^−1^ cornmeal, 20 g L^−1^ dextrose, 20 g L^−1^ agar) and synthetic low nutrient agar (SNA: 1.0 g L^−1^ KH_2_PO_4_, 10.5 g L^−1^ KCl, 1.0 g L^−1^ KNO_3_, 0.5 g L^−1^ MgSO_4_, 0.2 g L^−1^ dextrose, 0.2 g L^−1^ sucrose, 18 g L^−1^ agar), respectively. The characterization of conidium, conidiophore, chlamydospores and conidial pustules of *Trichoderma* strain cultured on the CMD and SNA mediums were examined after 3 and 7 days under optical microscope (Olympus, BX53F) and stereoscopic microscope (Olympus, SZX16), and compared with published morphology.

For molecular identification, the genomic DNAs were extracted using the CTAB method as described by Stewart and Via [27]. The partial translation elongation factor 1-alpha (*tef*1) gene and RNA polymerase subunit II (*rp*b2) gene were amplified using primer pairs EF1−728 F/TEF1LLErev and Frpb2-5f/Frpb2-7cr, respectively [28]. The PCR reaction mixture was as follows: 1× Rapid Taq Master Mix (Vazyme Biotech Co., Ltd., Nanjing, China), 0.2 μM Primer 1, 0.2 μM Primer 2, 100 ng gDNA, and the PCR conditions were: 94 °C for 5 min; followed by 30 cycles of 94 °C for 10 s, 55 °C for *tef*1 or 59 °C for *rpb*2 for 10 s, and 72 °C for 90 s; and 72 °C for 10 min. The PCR products were detected using 1.0 % (*w*/*v*) agarose gel electrophoresis, purified using ZTOPO-TA Fast Cloning Kit (Beijing Zoman Biotechnology Co., Ltd., Beijing, China, Kit No. ZC206-1), and sequenced by Guangzhou Tianyi Huiyuan Biotechnology Co., Ltd. (Guangzhou, China). The phylogenetic tree based on *tef*1 or *rpb*2 gene sequences was constructed using the neighbor-joining method in the MEGA-X program.

### 2.4. The Antagonistic Effect of the Combination of Hymexazol-Tolerant Trichoderma and Hymexazol to F. oxysporum

To evaluate the antagonism of the combination of hymexazol-tolerant *Trichoderma* and hymexazol to *F. oxysporum*, a dual culture of *Trichoderma* SC012 strain and *F. oxysporum* FC018 was conducted on a PDA medium mixed with different concentrations of hymexazol (0, 50, 100, 200, 300 and 400 µg mL^−1^) at 28 °C in darkness, and the strain *F. oxysporum* FC018 was cultured alone on a PDA medium with different concentrations of hymexazol used as negative control. Seven days later, the radius of *F. oxysporum* strain FC018 colonies was measured to calculate the inhibition rate according to the formula: inhibition rate % = ((the radius of pathogen colony of negative control—the radius of pathogen colony of treatment group)/the radius of pathogen colony of negative control) × 100% (Morton and Stroube 1955).

### 2.5. Analysis for Antagonistic Activity between Trichoderma and F. oxysporum

#### 2.5.1. Trypan Blue Staining

To determine the antagonistic activity of the pathogen at the interaction zone between *Trichoderma* and *F. oxysporum*, the trypan blue staining method was conducted [29]. *Trichoderma* SC012 strain and *F. oxysporum* FC018 strain were dual cultured on PDA dishes incubated at 28 °C in darkness for 7 days. Then, 5 mL 0.1% trypan blue dye solution was added into the plate to dye for 10 min at room temperature and the residual dye was removed with sterile water. The changes in mycelium staining and the interaction zone were observed under microscope and photographed.

#### 2.5.2. Effect of Volatile and Non-Volatile Metabolites Produced by *Trichoderma* SC012 Strain

The inhibitory effect of volatile and non-volatile metabolites produced by the *Trichoderma* SC012 strain against the *F. oxysporum* FC018 strain was evaluated by the method described by Dennis and Webster [30,31]. To test the effect of volatile metabolites, a cellophane sheet was sandwiched between two Petri dishes on which mycelium plugs of *Trichoderma* and *F. oxysporum* were inoculated, respectively. For non-volatile metabolites, *Trichoderma* was cultured on cellophane covering on the medium for two days and then *F. oxysporum* was inoculated on the medium after it was uncovered. The cultures were incubated at 28 °C in darkness for 7 days without the cellophane, and the colony radius of the pathogen was measured to calculate the inhibition ratio of mycelial growth. The formula of inhibition rates are as follows:

NT/N (%) = ((colony diameter of control without hymexazol − colony diameter of treatment group without hymexazol)/colony diameter of control without hymexazol) × 100%;

T/H (%) = ((colony diameter of control with hymexazol − colony diameter of treatment group with hymexazol)/colony diameter of control with hymexazol) × 100%;

T/N (%) = ((colony diameter of control without hymexazol − colony diameter of treatment group with hymexazol)/colony diameter of control without hymexazol) × 100%.

### 2.6. Trichoderma Population Dynamics in Rhizosphere Soil of Cowpea

The population dynamics of *Trichoderma* SC012 strain in the rhizosphere soil of cowpea were determined through the modified gnotobiotic tube system of Marco [32]. The soil was disinfected through dry heat sterilization at 180 °C for 6h, then autoclaved at 121 °C for 60 min for three consecutive days. The sterility of autoclaved soil was determined by the dilution plate technique. In aseptic conditions, the soil was placed into glass tubes (540 mm in length, 51 mm in inner diameter), and then the cowpea seeds which were surface-sterilized by sequential immersion in 75% (*v*/*v*) ethanol for 30 s and 8% (*w*/*v*) NaOCl for 1 min were planted in the soil, one seed per tube. The glass tubes were divided into four groups equally: the first group was irrigated with sterilized distilled water (50 mL per tube), the second group was irrigated with 100 µg mL^−1^ hymexazol (50 mL per tube), the third group was irrigated with 1 × 10^7^ cfu mL^−1^ *Trichoderma* SC012 strain spore suspension (50 mL per tube) and the last group was irrigated with the mixture of 5 × 10^6^ cfu mL^−1^ *Trichoderma* SC012 strain spore suspension and 50 µg mL^−1^ hymexazol (50 mL per tube). All tubes were incubated in a growth chamber (22–25 °C, 70% relative humidity, 12 h L/12 h D photoperiod) for 10 days. *Trichoderma* populations were re-isolated from the rhizosphere soil using the method of plate dilution [33] and incubated at 28 °C for 48 h. The colonies that appeared on the plates were counted as colony forming units (cfu g^−1^).

### 2.7. Greenhouse Experiments

The experiments were carried out in the Experimental Farm, Agricultural Science Base of College of Plant Protection, Hainan University (20°3′ N, 110°19′ E). The seeds of American non-bracket cowpea were surface-sterilized with 8% (*w*/*v*) NaOCl for 1 min, washed using distilled water three times, placed in plates with distilled water for germination and then the germinated seeds were sown in pots with 2 kg composite soil (soil: sand = 7: 3, 3 plants/pot). After 10 days, seedlings with uniform size were selected and equally divided into five groups. One group was irrigated with 30 mL distilled water as “blank control”, and the other four groups were irrigated with a 30 mL spore suspension of *F. oxysporum* (1 × 10^7^ cfu mL^−1^). Two days later, among the four groups irrigated with the spore suspension of *F. oxysporum*, one group was irrigated with sterilized distilled water (100 mL per pot) as “negative control”, the second group was irrigated with 100 µg mL^−1^ hymexazol (100 mL per pot) as “hymexazol treatment”, the third group was irrigated with 1 × 10^7^ cfu mL^−1^ spore suspension of *Trichoderma* SC012 strain (100 mL per pot) as “*Trichoderma* treatment” and the last group was irrigated with the mixture of 5 × 10^6^ cfu mL^−1^ *Trichoderma* SC012 strain spore suspension and 50 µg mL^−1^ hymexazol (100 mL per pot) as “combine treatment”. Before inoculated by spore suspension of pathogen, the cowpea roots were injured by inserting transplanting shovel into soil near the plant. Then, all groups were irrigated with water when needed for the next 20 days. All treatments were arranged in a completely randomized design (CRD) with at least 40 cowpea seedlings for each treatment. Twenty days after inoculation, the disease index and control effect were investigated according to the grading standard of Rigert and Foster [34]. Disease index = Ʃ (number of diseased plants × representative series)/(total number of plants × highest representative level value) × 100. Control effect (%) = (infected control disease index − treatment disease index)/infected control disease index × 100%.

### 2.8. Field Experiments

The field experiments were conducted at the Experimental Farm, Danzhou Campus, Hainan University (19°30′ N, 110°29′ E) for two independent times in June 2020 and June 2021. Field plots (10 m × 19.9 m) comprising 17 rows and 30 holes per row were arranged in a completely randomized block design and three cowpea seeds were sown in each hole. The treatments of the field experiment were the same as that of the greenhouse experiment, with three rows and at least 230 cowpea seedlings for each treatment, and the outside two rows serving as guard rows. All plants were harvested after 20 days and used to evaluate control effect.

### 2.9. Statistical Analyses

Statistical analyses of quantification data were carried out using IBM SPSS Statistics 21 (SPSS Inc, Chicago, IL, USA) software, and were subject to one-way analysis of variance (ANOVA) analysis. Means were analyzed using Tukey’s test and Dunnett’s *t*-test at *p* < 0.05. All experiments were repeated at least three times.

## 3. Results

### 3.1. The Sensitivity of Trichoderma SC012 Strain and F. oxysporum FC018 to Hymexazol

Among all the *Trichoderma* strains tested, the SC012 strain displayed the strongest tolerance to hymexazol (EC_50_ = 263.68 µg mL^−1^) and was chosen for further analysis (Table 1). The results also showed that hymexazol significantly suppressed the mycelial growth of *F. oxysporum* FC018 with an EC50 value being 62.15 µg mL^−1^.

### 3.2. Taxonomic Classification of Trichoderma SC012 Strain

**Figure 1:**
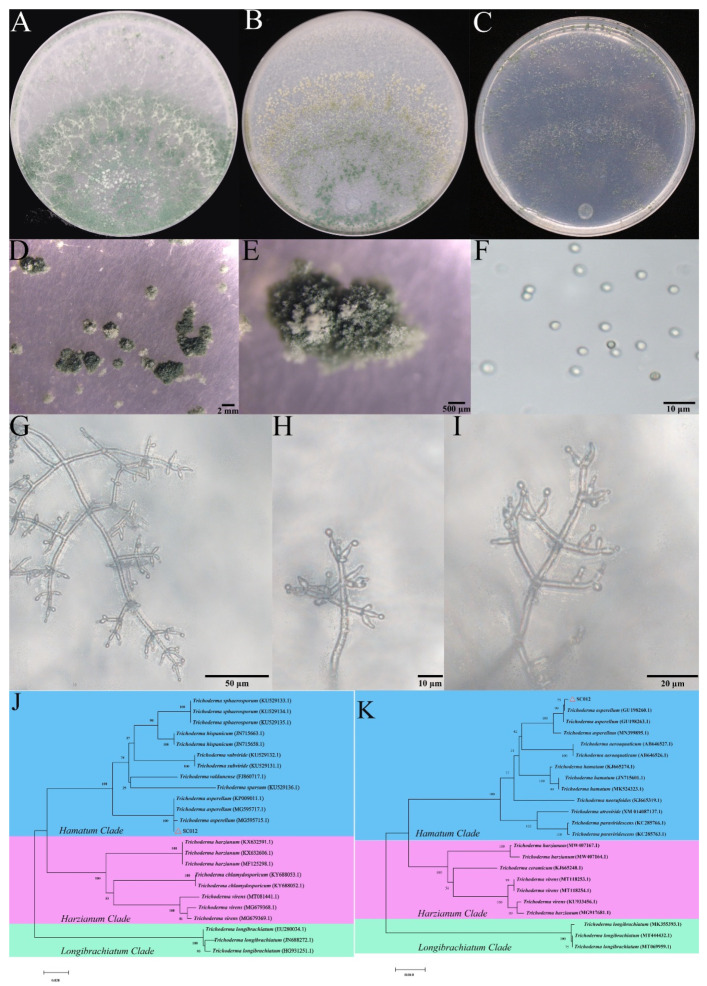
Morphology and molecular analysis of *Trichoderma* SC012 strain. (**A**–**C**) The morphology of colony grown on PDA, CMD and SNA medium, respectively; (**D**,**E**) the morphology of a conidial pustule grown on CMD plates; (**F**) morphology of the conidia and chlamydospores; (**G**–**I**) the morphology of conidiophores; (**J**) neighbor-joining tree based on translation elongation factor 1 (*tef*1) gene sequences and evaluated by 1000 bootstrap replications; (**K**) neighbor-joining tree based on RNA polymerase subunit II (*rpb*2) gene sequences and evaluated by 1000 bootstrap replications.

Seven day old culture of *Trichoderma* SC012 strain showed two or more concentric rings on PDA, CMD and SNA medium (Figure 1A–C) and there was no pigment or distinctive odor detected on PDA medium. On the CMD medium, loosely organized and dark green conidial pustules with a diameter of 1–2 mm were observed (Figure 1B,D,E). The branches of conidiophore were pyramidal with verticillate and paired lateral branches arising from main axis and rebranching, and all branches aroused at an angle of nearly 90° with the subtending branch (Figure 1G–I). The conidia were smooth and 2.5–3.0 × 3.0–4.0 μm in size, showing sub globose to ovoidal shape (Figure 1F). The morphology of SC012 was the same as *T. asperellum* described in Samuels et al. [35]. The partial *tef*1 and *rpb*2 genes amplified from *Trichoderma* SC012 strain were sequenced and deposited in GenBank receiving the accession numbers MW197094 and MZ753814. The phylogenetic tree based on *tef*1 gene sequences showed that *Trichoderma* SC012 strain was closely related to KP009011.1, MG595717.1 and MG595715.1 (*T. asperellum*) (Figure 1J), and the phylogenetic tree based on *rpb*2 gene sequences indicated that *Trichoderma* SC012 strain was intimately related to GU198260.1, GU198263.1 and MN399895.1 (*T. asperellum*) (Figure 1K). Therefore, the SC012 strain was identified as *T. asperellum* according to the morphological characterization and molecular analysis.

### 3.3. Antagonism of the Combination of Trichoderma and Hymexazol to F. oxysporum

**Figure 2:**
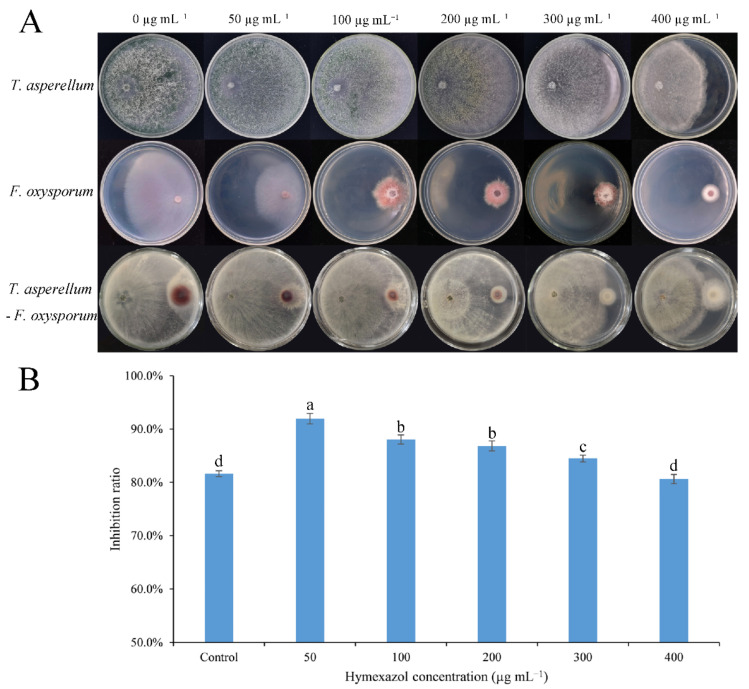
The antagonism effect of the combination of *Trichoderma* SC012 and hymexazol to *F. oxysporum* FC018. (**A**) Dual culture of *T. asperellum* SC012 and *F. oxysporum* FC018 at different concentrations of hymexazol; (**B**) inhibition rate of *Trichoderma* strain to *F. oxysporum* at different concentrations of hymexazol. Data presented are the means ± SE. The different lowercase letters indicate significant difference (*p* < 0.05) among the treatments.

The inhibition rate of *T. asperellum* SC012 against *F. oxysporum* FC018 was evaluated at different concentrations (0, 50, 100, 200, 300 and 400 µg mL^−1^) of hymexazol. Results showed that at 50 µg mL^−1^ of hymexazol, the inhibition rate of *T. asperellum* SC012 to *F. oxysporum* FC018 was the highest compared with control and other treatments (Figure 2). Therefore, 50 µg mL^−1^ of hymexazol was considered to have a better inhibition effect to *F. oxysporum* when combined with *T. asperellum* SC012 and was chosen for further analysis.

**Figure 3:**
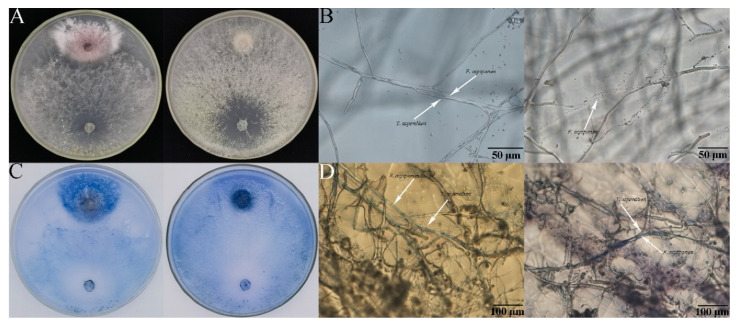
Observation of the interaction zone between *T. asperellum* SC012 and *F. oxysporum* FC018. (**A**), Dual culture of *T. asperellum* SC012 and *F. oxysporum* FC018 at the presence (right) or absence (left) of 50 µg mL^−1^ hymexazol. (**B**), Hyphae of *F. oxysporum* FC018 encircled (left) and disintegrated (right) by *T. asperellum* SC012. (**C**), Trypan blue staining of *T. asperellum* and *F. oxysporum* at the presence (right) or absence (left) of 50 µg mL^−1^ hymexazol. (**D**), Stained hyphae of *F. oxysporum*.

The hyphae of *F. oxysporum* FC018 were encircled and disintegrated by hyphae of *T. asperellum* SC012 strain at the interaction zone between these two fungi (Figure 3B). In the trypan blue staining experiment, the hyphae of *F. oxysporum* at the interaction zone were clearly stained blue, indicating that these hyphae had died (Figure 3C). Microscopic observation showed that unstained *T. asperellum* hyphae penetrated and encircled the stained *F. oxysporum* hyphae (Figure 3D).

**Figure 4:**
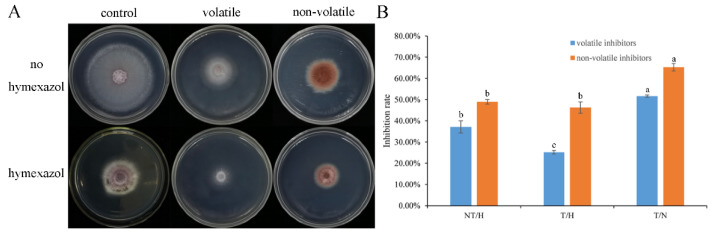
Effect of volatile and non-volatile metabolites produced by *T. asperellum* SC012 to *F. oxysporum* FC018 at the presence or absence of 50 µg mL^−1^ hymexazol. (**A**) *F. oxysporum* FC018 grown on PDA medium affected by volatile and non-volatile metabolites; (**B**) the inhibition rates of volatile and non-volatile metabolites to the growth of *F. oxysporum* FC018. Data presented are the means ± SE. The lowercase letters indicate significant difference (*p* < 0.05) among the treatments.

The volatile and non-volatile compounds produced by *T. asperellum* SC012 suppressed the growth of hyphae and the inhibition rates were higher than 40% (Figure 4). The inhibition rates of volatile and non-volatile compounds (“T/H”) decreased by 11.94% and 2.98% and “T/N” increased by 14.53% and 16.33% when compared with “NT/N”, respectively (Figure 4B). It seems that the combined application has greater effect on *F. oxysporum* growth.

### 3.4. Colonization of Trichoderma SC012 Strain in the Rhizosphere Soil of Cowpea

**Figure 5:**
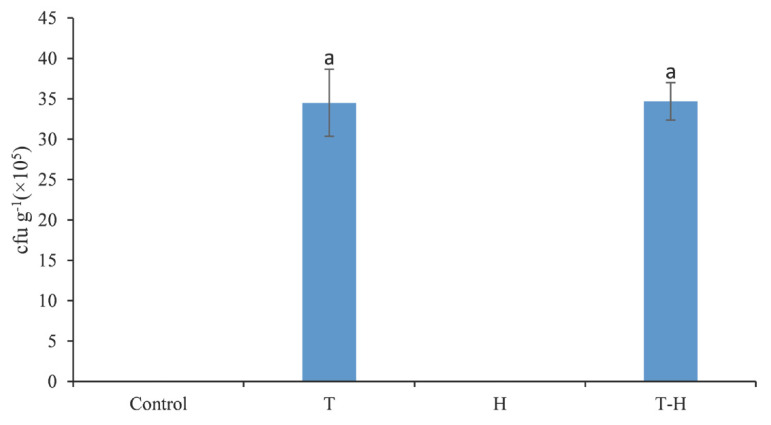
Colonization of *T. asperellum* SC012 strain in rhizosphere soil of cowpea. “Control”, “T”, “H” and “T-H” stand for “negative control”, “*Trichoderma* treatment”, “hymexazol treatment” and “combination treatment”, respectively. Data presented are the means ± SE (*n* = 3). Different letters indicate significant difference (*p* < 0.05) among the treatments.

*T. asperellum* SC012 strain could colonize well the rhizosphere soil of cowpea both in the presence or absence of hymexazol and no significant difference was found between these two treatments (Figure 5). The population density of *T. asperellum* SC012 strain was 3.45 × 10^6^ cfu g^−1^ when inoculated by *Trichoderma* alone and was 3.47 × 10^6^ cfu g^−1^ when the combination of *Trichoderma* and hymexazol was used (Figure 5). This showed that the combination of *Trichoderma* and hymexazol had no significant effect on the colonization of *T. asperellum* SC012 strain in the rhizosphere soil of cowpea.

### 3.5. The Combination of Trichoderma and Hymexazol Can Effectively Control Cowpea Fusarium Wilt in Greenhouse

**Figure 6:**
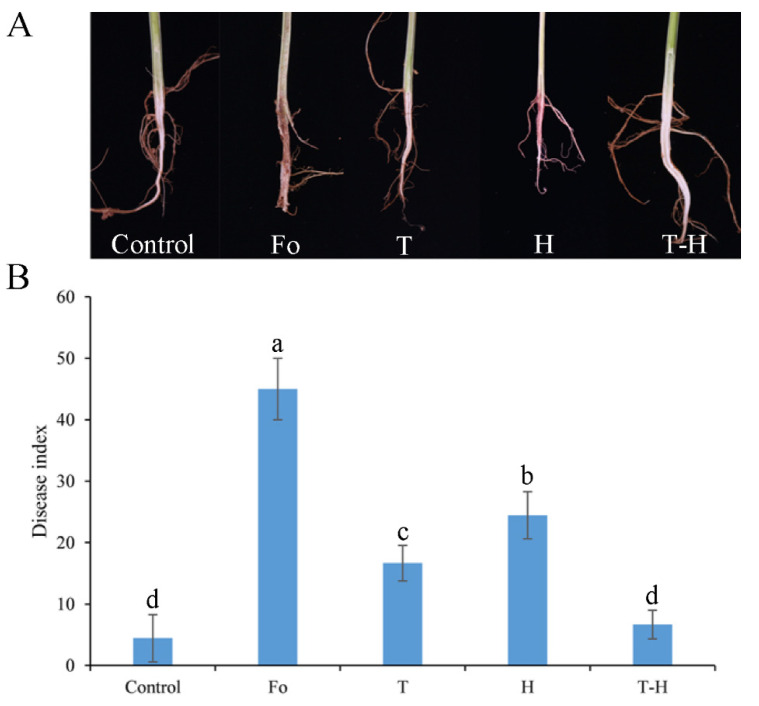
The effect of the combination of *T. asperellum* SC012 and hymexazol to control cowpea Fusarium wilt in greenhouse. (**A**) The symptoms of cowpea root; (**B**) the disease index of cowpea Fusarium wilt. “Control”, “Fo”, “T”, “H” and “T-H” stand for “blank control”, “negative control”, “*Trichoderma* treatment”, “hymexazol treatment” and “combination treatment”, respectively. Data presented are the means ± SE. Different letters indicate significant difference (*p* < 0.05) among the treatments.

The severity of symptoms of cowpea Fusarium wilt disease were significantly reduced when seedlings were treated separately with the *Trichoderma*, hymexazol and their combination preparation, which could be concluded from the different incidence of rot and browning of vascular bundles in cowpea roots under different treatments (Figure 6A). Additionally, also, the disease index of the group treated with the combination of *Trichoderma* and hymexazol was reduced significantly compared to other treatments (Figure 6B). The control effect of the group treated with *T. asperellum* SC012 alone was 62.69% and was 45.80% in the treatment with hymexazol alone. Conversely, for the treatment with the combination of *Trichoderma* and hymexazol, the control effect was 84.74%, which was significantly higher than other treatments (Figure 6B). The results indicated that this combination preparation had distinctive inhibitory effect on cowpea Fusarium wilt in greenhouse.

### 3.6. The Combination of Trichoderma and Hymexazol Can Effectively Control Cowpea Fusarium Wilt in Field

**Figure 7:**
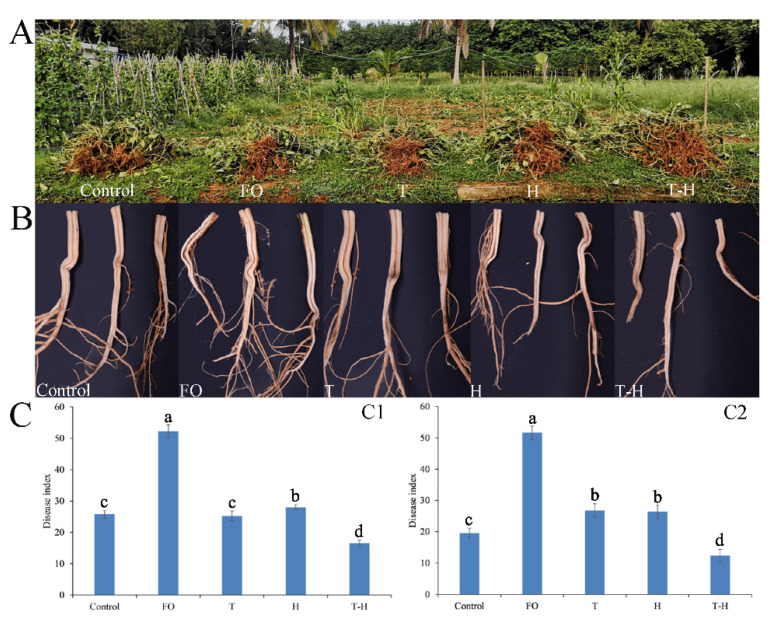
The effect of the combination of *T. asperellum* SC012 and hymexazol to control cowpea Fusarium wilt in fields. (**A**) The growth status of cowpea; (**B**) the symptoms of cowpea root; (**C**) the disease index of cowpea Fusarium wilt. (**C1**) and (**C2**) represent two field experiments conducted in June of 2020 and June of 2021, respectively. “Control”, “FO”, “T”, “H” and “T-H” stand for “blank control”, “negative control”, “*Trichoderma* treatment”, “hymexazol treatment” and “combination treatment”, respectively. Data presented are the means ± SE. Different letters indicate significant difference (*p* < 0.05) among the treatments.

The field experiments were conducted for two seasons in June 2020 and June 2021, respectively, and we found that the combination of *T. asperellum* SC012 and hymexazol reduced the cowpea wilt disease caused by *F. oxysporum* (Figure 7). The disease index of the groups treated with the combination of hymexazol and *Trichoderma* was reduced significantly compared to other treatments in the two field experiments (Figure 7B,C). The control effects of the groups treated with the combination of hymexazol and *Trichoderma* were 68.39% and 75.69%, respectively, and were significantly higher than the groups treated with *T. asperellum* SC012 or hymexazol. The disease severity was reduced by 51.62% (*Trichoderma* treatment) and 46.38% (hymexazol treatment) in the first field experiment and by 47.45% (*Trichoderma* treatment) and 48.24% (hymexazol treatment) in the second field experiment. The results showed that the combination of hymexazol and *T. asperellum* SC012 could inhibit cowpea Fusarium wilt effectively under field conditions.

## 4. Discussion

Previous studies have reported that fungicides combined with biocontrol agents can inhibit pathogens or improve disease resistance of plants [36,37]. Hymexazol can effectively inhibit the normal growth of fungal mycelium or directly kill pathogens of plants, and can promote the growth of plant roots, such as through rooting and seedling strengthening [38,39]. Myresiotis et al. [22] reported that the combination of plant-growth-promoting rhizobacteria and hymexazol could effectively control Fusarium crown and root rot on tomatoes. Abo-Elyousr et al. [21] demonstrated that *Trichoderma* combined with resistance inducers could control cotton root rot. However, few studies showed that the combination of *Trichoderma* and fungicide can prevent plant diseases [21,40]. In this study, the combined use of *T. asperellum* SC012 and hymexazol against cowpea wilt disease caused by *F. oxysporum* was investigated. Biocontrol fungus can be affected by chemical pesticides and the impact should be considered when they are applied together. In the present study, a hymexazol-resistant *T. asperellum* strain was selected and when hymexazol was combined at the concentration of 50 µg mL^−1^, the combination of *Trichoderma* and hymexazol enhanced antagonistic effects towards *F. oxysporum*.

Studies have shown that *T. asperellum* is particularly effective in controlling Fusarium wilt [41,42]. Our results demonstrated that the selected hymexazol-resistant *T. asperellum* SC012 strain showed high antagonistic activities (77.36%), inhibiting the mycelial growth of *F. oxysporum* FC018. The hyphae of *F. oxysporum* FC018 were penetrated, encircled and degraded by *T. asperellum* SC012, indicating that *Trichoderma* could parasitize the cowpea pathogenic fungi *F. oxysporum*. Previous studies have illustrated that *Trichoderma* species produce attachment and infection structures and kill parasitized fungi by cell wall-degrading enzymes (CWDEs), often in combination with the secretion of antimicrobial secondary metabolites which rely on G-protein signaling, the cAMP pathway, and MAPK cascades [13,14,18,43].

The colonization of rhizosphere microorganisms in plant roots plays an important role in plant nutrition, competition and disease resistance [44]. This symbiotic relationship could mildly and effectively activate the plant immune response locally or systemically, thus plants respond more effectively to the stresses of potential biotic and abiotic factors [45,46]. Moreover, the relatively poor biocontrol effect observed in previous studies was usually associated with the failure of the colonization of inoculated biocontrol bacteria in the rhizosphere [47]. In this study, the results indicated that the *T. asperellum* SC012 strain could successfully colonize the rhizosphere of cowpea even in the presence of hymexazol. Additionally, when the concentration of the *T. asperellum* SC012 spore was half in the mixture *Trichoderma*/hymexazol, the population density of *Trichoderma* in the rhizosphere soil did not differ significantly from the full-dose treatment with *T. asperellum* SC012 alone. We guessed that in this range of spore concentrations the reduction in the amount of inoculum of *T. asperellum* SC012 did not affect disease control effectiveness of *Trichoderma*. Therefore, further experiments are needed in order to investigate the colonizing ability of *T. asperellum* SC012 in the cowpea root system in natural soil.

In the greenhouse and field experiments, when *T. asperellum* SC012 and hymexazol were applied together they controlled cowpea wilt disease more effectively than when they were applied singularly, even when their dose was halved. In conclusion, the combination of *Trichoderma* and hymexazol can reduce the use of chemical fungicide, which offers the opportunity of more eco-friendly management strategies for the integrated control of Fusarium wilt of cowpea and the possibly of soil-borne diseases of other crops.

## Figures and Tables

**Table 1:** Inhibition activities of hymexazol on *Trichoderma* and pathogen *F. oxysporum*.

Taxonomic Status	Strains	EC50 (µg mL^−1^)
*Trichoderma*	SC012	263.68 ± 4.01 a
	LS007-21	192.02 ± 0.98 c
	HN082102.1	128.53 ± 3.16 d
	HL167	153.55 ± 3.51 e
	DQ1	226.01 ± 2.65 b
*F. oxysporum*	FC018	62.15 ± 1.78 f

Note: Data presented are the means ± SE. The lowercase letters indicate significant difference (*p* < 0.05) among the treatments.

## Data Availability

Not applicable.
